# Filarial pleural effusion

**DOI:** 10.4103/1817-1737.74279

**Published:** 2011

**Authors:** K. Gowrinath

**Affiliations:** *Department of Pulmonary Medicine, Narayana Medical College, Nellore-524002, India. E-mail: drkgowrinath@gmail.com*

Sir,

I read the article of Garg, *et al*.[1] with great interest. As a co-author of our work[2] cited in their article, I would like to make it clear that the microfilariae detected in the pleural biopsy material of our case were of *Wuchereria bancrofti* and not *Mansonella perstans* as quoted in the text by the authors. I was surprised to note that the closed pleural biopsy was not carried out as there was a chance to establish the filarial etiology within the pleura. Microfilariae reside in the arterioles of pulmonary system during daytime and appear in peripheral blood and other body fluids only in the night time during the peak biting time of mosquito vectors. The traditional diagnostic method of filariasis is to demonstrate microfilariae microscopically in the peripheral blood (capillary finger prick or thick venous blood smears) drawn in the night or presence of circulating filarial antigen.[3] Filariasis is a major health problem in India and microfilariae have been detected along with other diseases such as tuberculosis, non-Hodgkin’s disease, etc.[4] I am curious to know the scientific basis regarding the number of times a clinical specimen like pleural fluid can be tested and methodology adapted by the authors who successfully demonstrated microfilariae on all four occasions to conclude that the pleural effusion was due to filariasis only.
